# Hepatitis B virus X protein modulates apoptosis in human renal proximal tubular epithelial cells by activating the JAK2/STAT3 signaling pathway

**DOI:** 10.3892/ijmm.2013.1295

**Published:** 2013-03-07

**Authors:** PING HE, DAN ZHANG, HONG LI, XU YANG, DETIAN LI, YONGZHEN ZHAI, LI MA, GUOHE FENG

**Affiliations:** 1 Department of Nephrology, Shengjing Hospital of China Medical University, Shenyang, Liaoning 110004, P.R. China; 2 Department of Infectious Diseases, Shengjing Hospital of China Medical University, Shenyang, Liaoning 110004, P.R. China; 3 Department of Cardiology, Shengjing Hospital of China Medical University, Shenyang, Liaoning 110004, P.R. China

**Keywords:** apoptosis, HBV X protein, JAK2/STAT3 signaling pathway, HK-2 cells, AG490

## Abstract

Hepatitis B virus X protein (HBx) is a multifunctional protein, and it activates multiple signal transduction pathways in multiple types of cells and regulates the process of cell apoptosis. In the present study, we mainly investigated the correlation between HBx and renal tubular epithelial cell apoptosis in hepatitis B virus-associated glomerulonephritis (HBVGN) and the possible signaling mechanism. Cell apoptosis in nephridial tissues of patients with HBVGN were determined by the TUNEL method. HBx, p-STAT3 and STAT3 levels in nephridial tissues were determined by immunohistochemical assay, and a correlation analysis between HBx expression levels and apoptosis index in nephridial tissues was conducted. The activation of the JAK2/STAT3 signaling pathway in HK-2 cells and the expression of the apoptosis-related proteins Bax and Bcl-2 were determined by western blot analysis following transfection with the HBx eukaryotic expression vector. Cellular proliferation activity was determined by the CCK-8 method, and cell apoptosis was determined with HO33342 staining using transmission electron microscopy and Annexin V/PI double staining flow cytometry. The results revealed that the apoptosis index in nephridial tissues of patients with HBVGN was significantly higher when compared to that of the control group, and p-STAT3 expression levels in HBVGN nephridial tissues were significantly increased. In the control group, no HBx expression was observed in the nephridial tissues, whereas HBx expression was found in the nephridial tissues of 86% of the patients with HBVGN. The HBx expression levels had a linear correlation with the apoptosis index in the nephridial tissues. After target gene HBx infection, expression levels of both p-JAK2 and p-STAT3 in human proximal HK-2 cells were significantly increased, and the Bax/Bcl-2 ratio was also significantly increased. At the same time, cellular proliferation of HK-2 cells was significantly inhibited, and the rate of apoptosis was increased. After incubation with AG490, the JAK2/STAT3 signaling pathway was partially blocked, which caused a decrease in the Bax/Bcl-2 ratio and reduced cell apoptosis caused by HBx. In conclusion, HBx upregulates the Bax/Bcl-2 ratio by activating the JAK2/STAT3 signaling pathway to cause renal tubular epithelial cell apoptosis, and it is possibly involved in the pathogenic mechanism of nephridial tissue damage caused by HBV.

## Introduction

Hepatitis B virus (HBV) infection is a significant public health problem worldwide. It is estimated that 350 million individuals are chronic HBV carriers worldwide (1). For a long time, China, Southeast Asia and Tropical Africa have belonged to highly endemic areas. Since Combes *et al*(2) first described the first case of a patient with persistent HBsAg viremia suffering from membranous nephropathy (MN) in 1971, glomerulonephritis has been recognized as the most common extrahepatic lesion caused by HBV infection, and is also called HBV-associated glomerulonephritis (HBVGN). It was previously thought that the pathogenesis of HBVGN lay in the deposition of circulating immune and *in situ* immune complexes formed by HBV antigen and antibody on nephridial tissue. However, it is currently believed that HBV directly infects nephridial tissue cells due to its wide tropism to generate a viral cytocidal effect, which is also one of the important pathogeneses of HBVGN (3).

The HBV X protein (HBx) gene is the smallest open reading frame in the HBV genome, and it is located at 1374–1838 bp of the HBV genome. The overall length is 435 to 462 bp, and the code length is of a protein containing 154 amino acids. The X protein is a multifunctional protein and it activates multiple cellular signal transduction pathways and regulates apoptosis. However, the effects and mechanisms of HBx concerning the regulation of cell apoptosis vary in different types of cells and in different external conditions (4). A number of studies suggest that HBx can activate signaling pathways of JAK/STAT, Ras-Raf-MAPK, p38MAPK, JNK, P13K, Src tyrosine kinase and Pyk-2 (5,6) to induce host cell apoptosis (7–9).

Cell apoptosis is one type of cellular initiative death that occurs according to a certain procedure under gene control and enzymatic reactions. Aspartic acid cysteine protease-3 (caspase-3) is the final effector enzyme for apoptosis generation. Apoptosis-related proteins Bcl-2 and Bax are upstream substances of caspase-3. Among them, Bcl-2 is an anti-apoptosis protein, whereas Bax is contrary to Bcl-2 and is a typical pro-apoptosis protein. Therefore, expression levels of Bax and Bcl-2 and the Bcl-2/Bax ratio are important factors influencing cell survival (10–12). Expression of Bcl-2 and Bax is regulated by the JAK/STAT signaling pathway (13). The JAK/STAT signaling pathway is an important cytokine signal transduction pathway, and it is closely related to cellular proliferation, differentiation and apoptosis. JAK is one type of endogenous protein tyrosine kinase. After the cytokine receptor binds with corresponding aglucon, it can be activated to cause phosphorylation of the STAT molecule in the cytoplasm. Two phosphorylated STAT molecules form a dimer to enter the nucleus, and they bind with a specific DNA sequence of the target gene promotor in the nucleus to induce target gene expression. Among them, STAT tyrosine phosphorylation is the key link of the JAK/STAT signaling pathway regulating transcription and exerting multiple biological effects. Studies suggest that the occurrence and development of multiple acute and chronic kidney diseases are closely related to cell apoptosis (14–17). Previous studies on HBVGN nephridial tissues showed that the main sites of occurrence of cell apoptosis were in proximal and distal renal tubular epithelial cells, rarely in the renal glomerulus. At the same time, the ratio of apoptosis-related proteins Bax/Bcl-2 was correspondingly altered. However, JAK/STAT signaling pathway activation and its role in cell apoptosis are still unclear (18–20).

In order to further clarify the pathogenic mechanism of the HBx gene in HBVGN and the role of JAK/STAT signaling pathway activation in renal tubular epithelial cell apoptosis, we conducted a correlation analysis of renal tubular epithelial cell apoptosis and HBx expression in renal biopsy tissues of 22 patients with HBVGN. We transfected renal tubular epithelial cells (HK-2) with the eukaryotic expression vector of the HBx gene to determine the activation of the JAK/STAT signaling pathway and its influences on proliferation and apoptosis of HK-2 cells in order to provide new experimental data for research on the molecular mechanisms of HBVGN.

## Subjects and methods

### Subjects

The subjects enrolled for the clinical pathological study were divided into 2 groups: the HBVGN group and the control group. The HBVGN group, consisted of 22 patients with HBVGN diagnosed by renal puncture biopsy at the Renal Department of the Shengjing Hospital Affiliated to China Medical University (Shenyang, China) from November, 2004 to March, 2011. Patients included 17 males and 5 females with ages ranging from 15 to 57 years and a mean age of 42.62±2.86 years. Serologic detection for HBV confirmed that all cases presented HBV infection, and pathological examination of nephridial tissues obtained by percutaneous renal biopsy under the guidance of B ultrasound confirmed that all cases presented with HBVGN. The control group consisted of 5 cases, including 4 males and 1 female. All sample tissues were from paraffin blocks of normal nephridial tissues of the farthest section margin to the lesion obtained following renal trauma operation or renal cancer surgery at our hospital. Serologic detection of HBV confirmed that all control cases had no HBV infection. This study was conducted in accordance with the Declaration of Helsinki. This study was conducted following approval from the Ethics Committee of Shengjing Hospital Affiliated to China Medical University. Written informed consent was obtained from all participants.

### Plasmid construction

Full-length HBx was PCR amplified from the p1.2II plasmid (HBV adr genome). The primers were: forward, 5′-gcgaattcatggctgctagggtgtgct-3′ and reverse, 5′-atctcgagttaggcagaggtgaaaaagttgc-3′, and were synthesized by Takara Co. (Dalian, China) and inserted into vector pcDNA3.1(+) (Invitrogen, USA). The PCR amplification protocol consisted of an initial 4-min denaturation at 94°C; followed by 25 cycles of denaturation at 94°C for 1 min, annealing at 60°C for 1 min, and extension at 72°C for 1 min; followed by a final extension at 72°C for 10 min. All ligated vectors were confirmed by DNA sequence analysis.

### Immunohistochemistry

The nephridial tissue paraffin sections (~3 μm) were dewaxed and placed into water. According to the kit instructions, the procedure was conducted. The primary antibodies: rabbit anti-HBx monoclonal (1:200) (Chemicon Co.), mouse anti-STAT3 monoclonal (1:120) (#9139), rabbit anti-p-STAT3 (1:150) (#9145) (both from Cell Signaling Technology), rabbit anti-Bcl-2 and rabbit anti-Bax (Santa Cruz Biotechnology, Inc., Santa Cruz, CA, USA) were respectively added and incubated at 4°C overnight. Subsequently, the biotin-labeled corresponding secondary antibody was added and incubated at 4°C for 20 min. The sections were washed with PBS, developed with DAB and mounted. In addition, a semi-quantitative analysis was conducted for the immunohistochemistry images. For each image, 20 visual fields were randomly selected, and the Olympus image pick up system and Japan MetaMorph/C-5050/BX41 mode microscopic image analysis system were used to determine the optical density value.

### TUNEL assay

The nephridial tissue paraffin sections (~3 μm) were dewaxed and placed into water. Digestion was conducted with 20 μg/ml protease K at room temperature for 20 min. Staining, developing and mounting were conducted successively according to the steps provided by the kit (*In Situ* Cell Detection Kit, POD; Roche). In addition, the sections digested without protease K were set as the control. For calculation of the apoptosis index (AI), 20 high-magnification visual fields (x400) were randomly selected from each section, and the number of TUNEL-positive cells were counted in each high-magnification visual field to respectively determine the mean percentage of positive cells in each high-magnification visual field. At last, comparisons were conducted for the mean values of the various groups.

### Cell culture, grouping and plasmid transfection

Human proximal HK-2 cells were purchased from Beijing Zhongyuan Co., Ltd. (ATCC distributor, China) and cultured in keratinocyte-SFM culture medium in an incubator containing 5% carbon dioxide at 37°C at a saturated humidity. Cells grew by attaching to the wall in a monolayer, and the cells in the logarithmic growth phase were used for experiments. After the cells covered the entire culture bottle bottom (generally 2 to 3 days), digestion and passage were conducted with 0.25% pancreatin under aseptic conditions.

The cell experiment included two parts. The first part included five groups: i) the control group, ii) empty plasmid pc-DNA3.1(+) transfection group (referred to as pc-DNA3.1(+) group), iii) the group transfected with plasmid pc-DNA3.1(+) HBx for 24 h (referred to as the HBx 24 h group), iv) the group transfected with plasmid pc-DNA3.1(+) HBx for 48 h (referred to as the HBx 48 h group), v) the group transfected with plasmid pc-DNA3.1(+) HBx for 72 h (referred to as the HBx 72 h group). The second part included 6 groups: i) the control group, ii) the pc-DNA3.1(+) group, iii) the HBx transfection group, iv) the control group with AG490, v) the pc-DNA3.1(+) group with AG490, vi) the HBx transfection group with AG490. For groups iv, v and vi, cells were incubated in AG490 (Sigma, USA) for 24 h.

Lipofectamine™ LTX and PLUS™ transfection reagents (Invitrogen, USA) were used to conduct the transfection according to the steps described in the kit instructions. In the experimental process, pEGFP-C1 fluorescence plasmid (Clontech) with a size similar to pcDNA3.1(+) HBx was used for cotransfection in order to indirectly evaluate the transfection efficiency of the target plasmid. The transfection efficiency was ~45–55%.

### Western blot analysis

After the cells were harvested, total cell protein was extracted with RIPA lysate containing PMSF. The protein concentration of the cell extract was determined by the BCA method. After an equivalent amount of the sample was added, electrophoretic separation was conducted respectively on 8 and 12% SDS-PAGE gel. Subsequently, the protein was transferred onto a PVDF membrane. After mounting, the primary antibodies: HBx (1:1,000), JAK2 (1:2,000), p-JAK2 (1:1,500), STAT3 (1:1,000), p-STAT (1:2,000) were respectively added and incubated in a table concentrator at 4°C overnight. Next, the secondary antibody was added to carry out the ECL reaction. Fixing and developing were conducted at one time, and image analysis system was used to determine the gray value of the band.

### Cell morphological analysis of apoptosis

Morphological variations of the apoptotic cells were observed by staining the nuclei with HO33342 (Sigma). HK-2 cells were inoculated into a 6-well plate at a density of 5×10^5^ cells/well and incubated overnight, and then transfected with the empty plasmid pc-DNA3.1(+) or pc-DNA3.1(+) HBx, respectively. At the same time, the control group was set. The cells were incubated in the incubator containing 5% carbon dioxide at 37°C at saturated humidity, respectively, for 24, 48 and 72 h. Subsequently, the cell slides were taken out, washed with PBS for three times, fixed with 4% paraformaldehyde for 20 min and washed with PBS again for three times. After HO33342 fluorochrome (5 mg/l) was added, the cell slides were incubated for 8 min at 37°C (protected from light) and rewashed with PBS three times. Observations and imaging were immediately conducted under a fluorescence microscope.

The ultrastructures of HK-2 cells were observed under a transmission electron microscope. Cells were inoculated into a 6-well plate (5×10^5^ cells/well) and respectively transfected with empty plasmid pc-DNA3.1(+) or pc-DNA3.1(+) HBx. At the same time, the control group was set. After the cells were incubated in the incubator containing 5% carbon dioxide at 37°C at saturated humidity respectively for 24, 48 and 72 h, the cells were digested with pancreatin and collected. The collected cells were fixed in 2.5% glutaraldehyde (stored at 4°C), adequately washed with 0.1 mol/l phosphate buffer solution, fixed with 1% osmic acid (OsO_4_) for 24 h, washed, dehydrated in gradient ethanol, replaced with acetone, soaked, embedded with epoxy resin Epon812, polymerized and sectioned with an LKB Microtome (Sweden) into ultrathin slices. At last, the ultrastructures of the renal tubular epithelial cells were observed using transmission electron microscopy.

### Annexin V/propidium iodide staining assay

Annexin V-FITC/PI flow-type double staining kit was purchased from Nanjing KGI Biotechnology Development Co., Ltd., China. HK-2 cells were inoculated into a 6-well plate (5×10^5^/ml) and cultured for 24 h. After cells attached to the wall, they were respectively transfected with empty plasmid pc-DNA3.1(+) or pc-DNA3.1(+) HBx. At the same time, the control group was set. After the cells were incubated in the incubator containing 5% carbon dioxide at 37°C at a saturated humidity respectively for 24, 48 and 72 h, the cells were digested with pancreatin without EDTA and collected. The collected cells were centrifuged, washed with PBS for three times and suspended in 500 μl binding buffer (1X) to form a cell suspension with a concentration of ~1×10^6^ cells/ml. After 5 μl Annexin V-FITC was added, the cell suspension was gently mixed well and incubated in a refrigerator at 4°C for 30 min (protected from light). Subsequently, 5 μl PI was added, and the resulting cell suspension was gently mixed well and incubated in a refrigerator at 4°C for 15 min (protected from light). Immediately, cell testing was conducted using flow cytometetry (within 1 h at latest), and analysis of data was carried out by CellQuest Professional software. Annexin V-FITC/PI double-labeling flow cytometry differentiates the normal, necrotic and apoptotic cells in a sample. With FITC and PI fluorescence as dual parameter point diagram, cells were divided into four quadrants: left lower quadrant (Annexin V-FITC^−^ and PI^−^, representing normal live cells), left upper quadrant (Annexin V-FITC^−^ and PI^+^, representing cells with mechanical damages), right lower quadrant (Annexin V-FITC^+^ and PI^−^, representing early apoptotic cells) and right upper quadrant (Annexin V-FITC^+^ and PI^+^, representing advanced apoptotic/necrotic cells). Moreover, the cell apoptosis rates of the various cell groups were respectively calculated, and comparisons of the apoptosis rates were conducted among the various groups.

### Determination of cellular proliferation

The effect of the inhibition of the HBx gene on the proliferation of HK-2 cells was determined by CCK-8 (Nanjing KGI Biological Technology Development Co., Ltd.). After cells in the logarithmic growth phase were digested and collected and prepared into a cell suspension with a concentration of 1×10^5^/ml, cell suspension was inoculated into three 96-well plates (100 μl/well) and incubated overnight. Observations under a microscope confirmed that the cells attached to the wall of the well. In each 96-well plate, the cell suspension was divided into three groups: the control group, the pc-DNA3.1(+) group and the pc-DNA3.1(+) HBx transfection group. In addition, the blank group (only full culture medium, without cells) was set, and the blank was used for zero calibration during colorimetric determination. The cells were incubated in the incubator containing 5% carbon dioxide at 37°C at a saturated humidity respectively for 24, 48 and 72 h. At 2 h before culture completion CCK-8 (10 μl) was added into each well, and the cells were continuously cultured at 37°C for 2 h. At last, the optical density value (OD) for each well was determined with an ELISA reader at 450 nm. The experiment was conducted in triplicate. The cellular survival rate and proliferation inhibition ratio were calculated according to the following formulas:

$$\begin{array}{l} \text{Survival}\;\text{rate }(\%)=(\text{OD value of the test group}/\text{OD value of the control}\;\text{group})\times 100\\ \text{Inhibition ratio}=(1-\text{survival rate})\times 100. \end{array}$$

### Immunofluorescence

As the various groups of cells grew and fused by 95–100% on coverslips, the cells was taken out from the incubator, washed with PBS, fixed with 4% formaldehyde, permeabilized with 0.5% Triton X-100 at 37°C for 20 min and sealed with serum. Subsequently, the primary antibodies, STAT3 (1:50) and p-STAT3 (1:100), were respectively added, and the cells were cultured at 4°C overnight. After the secondary antibody was added, the cells were re-stained with DAPI and mounted with 95% glycerol. Observations and imaging were conducted under a confocal fluorescence microscope.

### Immunocytochemistry

As various groups of cells grew and fused by 95–100% on coverslip, the cells was taken out from the incubator, washed with PBS, fixed with 4% formaldehyde, permeabilized with 0.5% Triton X-100 at 37°C for 20 min and sealed with serum. Subsequently, the primary antibodies, Bcl-2 (1:160) and Bax (1:320), were respectively added, and the cells were cultured at 4°C overnight and then washed with PBS. After the secondary antibody was added, biotinidase was added, and DAB development, hematoxylin re-staining, returning to blue, dehydration, permeabilizing, mounting and observation were conducted successively.

### Statistical analysis

Experimental data are expressed as means ± standard deviation, and statistical processing was conducted using SPSS13.0 statistical software. One-way ANOVA or t-test of comparison between two samples was used for comparison between groups, and a linear correlation test was used for correlation analysis. P<0.05 was considered to indicate a statistically significant difference.

## Results

### HBx, STAT3 and p-STAT3 expression and the Bax/Bcl-2 ratio

**Figure 1:**
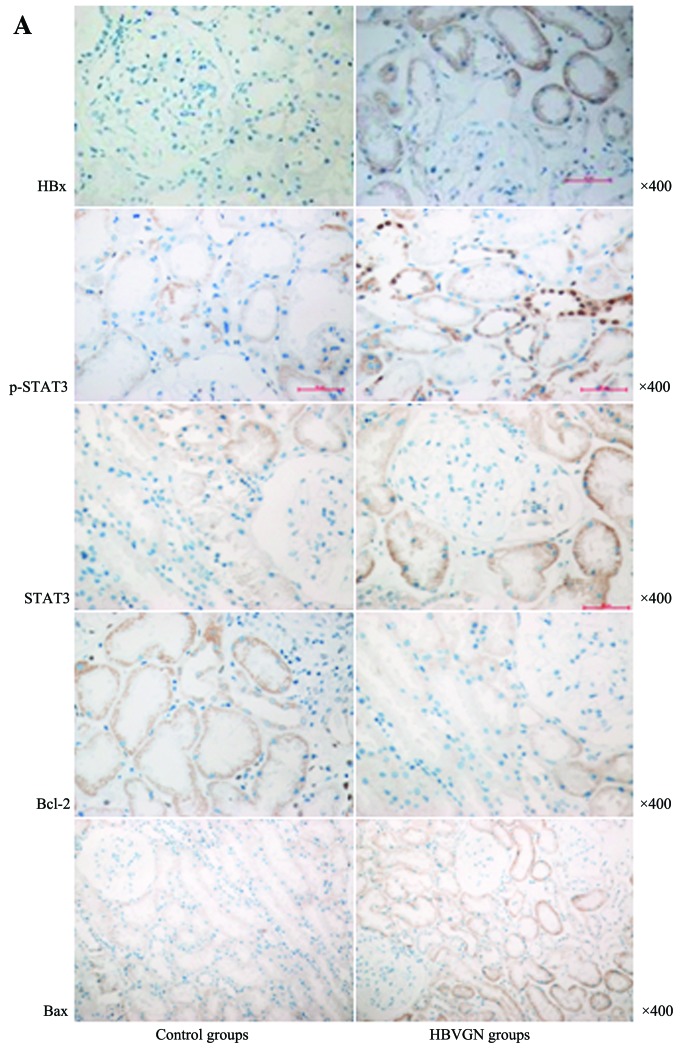

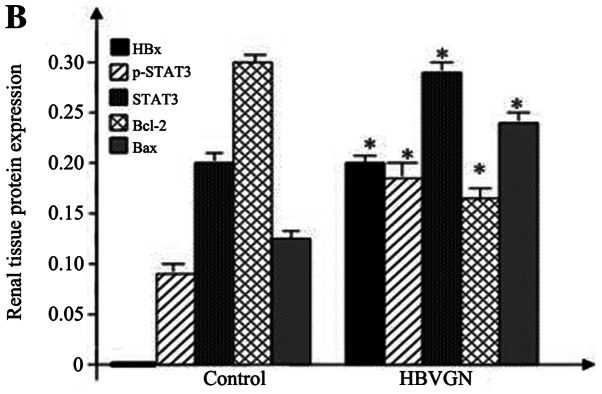
(A) HBx, STAT3, p-STAT3, Bcl-2 and Bax protein expression in nephridial tissues of the HBVGN patients and the control groups. (B) Histogram of the relative expression of HBx, STAT3, p-STAT3, Bcl-2 and Bax in the nephridial tissues of the control and the HBVGN patient groups.

HBx expression was noted in the renal tubular epithelial cells in 86% (19/22 cases) of the patients with HBVGN, and the integral optical density value was 0.24±0.03. It was evident under a microscope that the cytoplasm of the renal tubular epithelial cells presented brown and dark brown granules. HBx expression was not noted in the control group, while slight p-STAT3 expression (0.08±0.02) was evident mainly in the cytoplasm of the renal tubular epithelial cells. In the HBVGN group, p-STAT3 expression was slightly increased (0.17±0.04), and p-STAT3 expression was noted in both the cytoplasm and nucleus. Regarding the p-STAT3 expression in the nucleus, it was mainly present in the epithelial cell nuclei of proximal convoluted tubules and epithelial cell nuclei of collecting tubes, and there was a significant difference between the two groups (P<0.05). In both the control and HBVGN groups, STAT3 expression was found in the renal tubular epithelial cell cytoplasm. STAT3 expression in the HBVGN group was 0.32±0.04 and was higher than that of the control group (0.18±0.03). Between the two tissue groups, there was a significant difference (P<0.05). The optical density value for Bcl-2 in the control group was 0.34±0.05, and was mainly located in the renal tubular epithelial cell cytoplasm. Bcl-2 expression in the HBVGN group (0.16±0.04) was significantly lower than that in the normal group, and there was a significant difference between the two tissue groups (P<0.05). In the control group, Bax expression was lower (0.11±0.03), and Bax expression in the HBVGN group was slightly increased (0.26±0.04), mainly in the renal tubular epithelial cell cytoplasm. Between the two groups, a significant difference was achieved (P<0.05) (Fig. 1).

### Apoptosis index of the nephridial tissues

**Figure 2:**
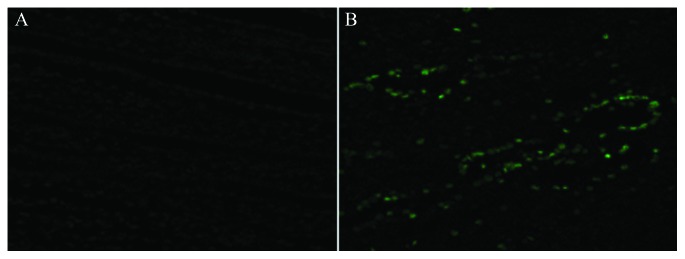
TUNEL-positive cells in nephridial tissues observed using confocal microscopy (x400).

For all 22 patients in the HBVGN group, TUNEL-positive cells were visible in the renal tubulointerstitial region, and the apoptosis index (AI) was 16.82±3.35%; TUNEL-positive cells were rare in the renal glomerulus. In the control group, a TUNEL-positive signal was occasionally visible in individual renal tubular epithelial cells, and the AI was 1.06±0.43%. Between the two groups, a significant difference for AI (P<0.05) was achieved (Fig. 2). In the HBVGN group, the AI was significantly correlated with renal HBx expression (r=0.612, P=0.032), and the AI was significantly correlated to proteinuria at 24 h (r=0.824, P=0.020). In addition, the AI in the renal tubules of patients with proteinuria content >3.5 g/day at 24 h was significantly higher when compared with the AI of patients with proteinuria content <3.5 g/day at 24 h.

### Target gene HBx expression

**Figure 3:**
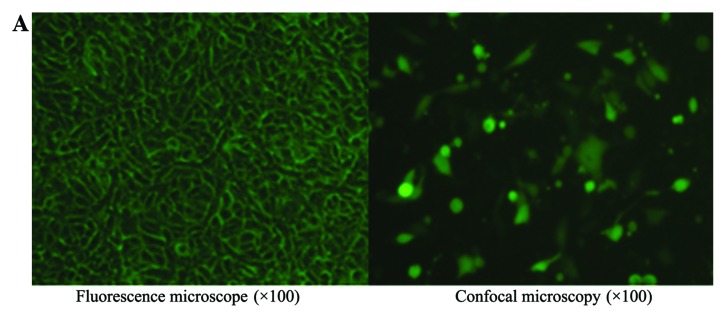

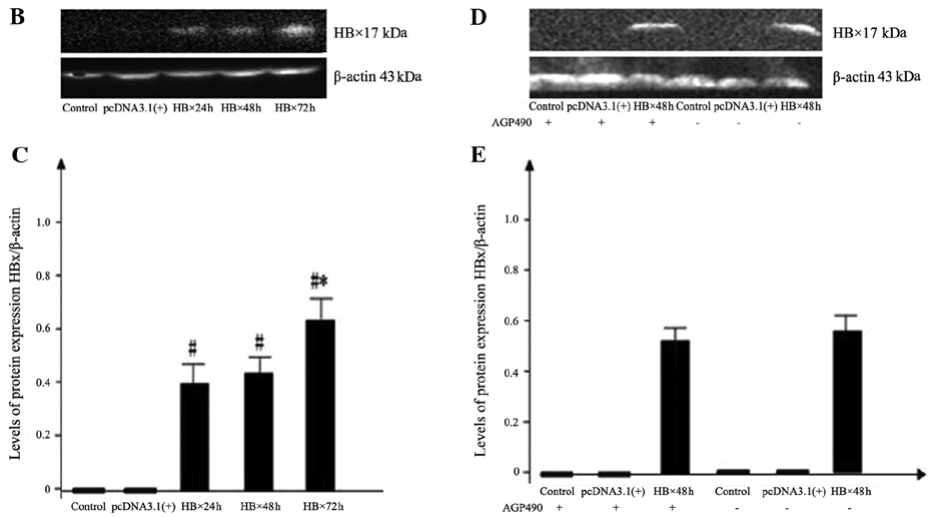
(A) Plasmid transfection efficiency as observed using confocal microscopy. (B) Western blot bands of HBx protein expression in the control group, the pc-DNA3.1(+) group, the HBx 24 h group, the HBx 48 h group and the HBx 72 h group. (C) Histogram of the relative expression of HBx protein in the control group, the pc-DNA3.1(+) group, the HBx 24 h group, the HBx 48 h group and the HBx 72 h group. (D) Western blot bands of HBx protein expression in the various groups with AG490, compared with the corresponding groups without AG490. (E) Histogram of the relative expression of HBx protein in the various groups with AG490, compared with the corresponding groups without AG490.

Confocal microscopy was used to observe the transfection efficiency of the pEGFP-C1 fluorescence plasmid cotransfected with pcDNA3.1(+) HBx in human proximal HK-2 cells. The transfection efficiency was ~45–55% (Fig. 3A). In the HBx 24 h group, the HBx 48 h group and the HBx 72 h group, there was an obvious band at the expected 17 kDa, respectively, suggesting that the HBx gene was expressed in the HK-2 cells. Between the HBx 24 h group and the HBx 48 h group, there was no significant difference in protein expression (P>0.05). Protein expression in the HBx 72 h group was highest and it was significantly higher than expression in the HBx 24 h and the HBx 48 h groups (both P<0.05). In the empty plasmid tranfection group and the control group, the corresponding band was not evident (Fig. 3B and C).

After the control group, the empty plasmid group and HBx transfection group were respectively transfected for 24 h, AG490 was added, and the incubation was continued for a further 24 h. HBx expression was evident in both the HBx transfection group and the HBx transfection with AG490 group, and a significant difference in HBx expression was noted (P>0.05), suggesting that AG490 did not influence HBx protein expression level (Fig. 3D and E).

### JAK2/STAT3 signaling pathway

**Figure 4:**
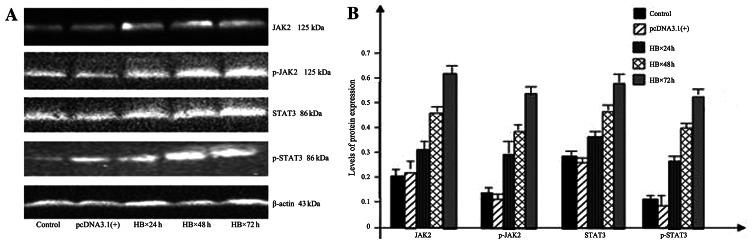

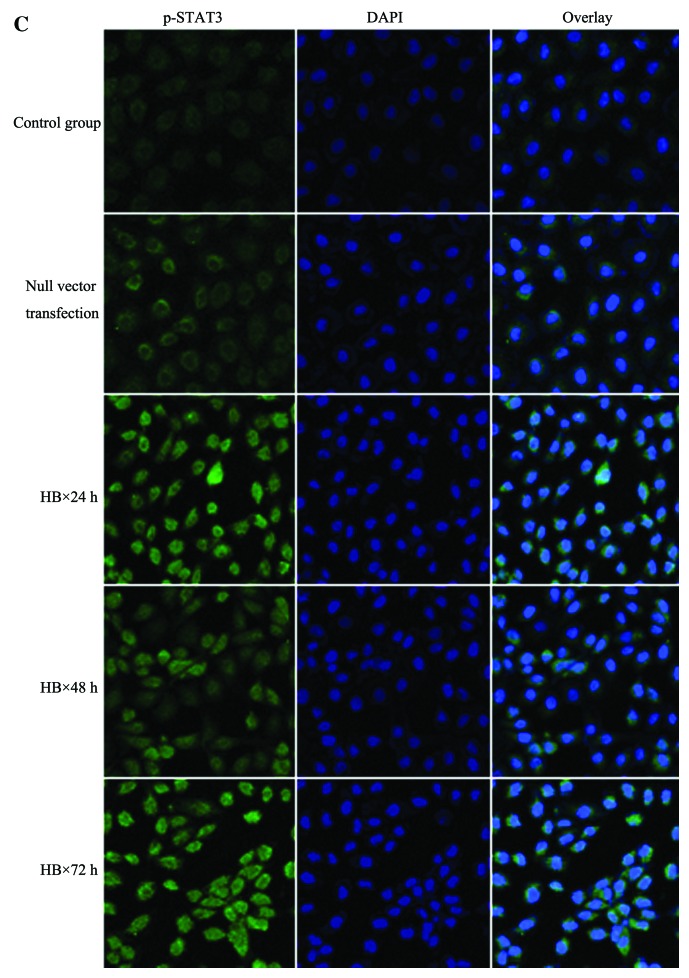

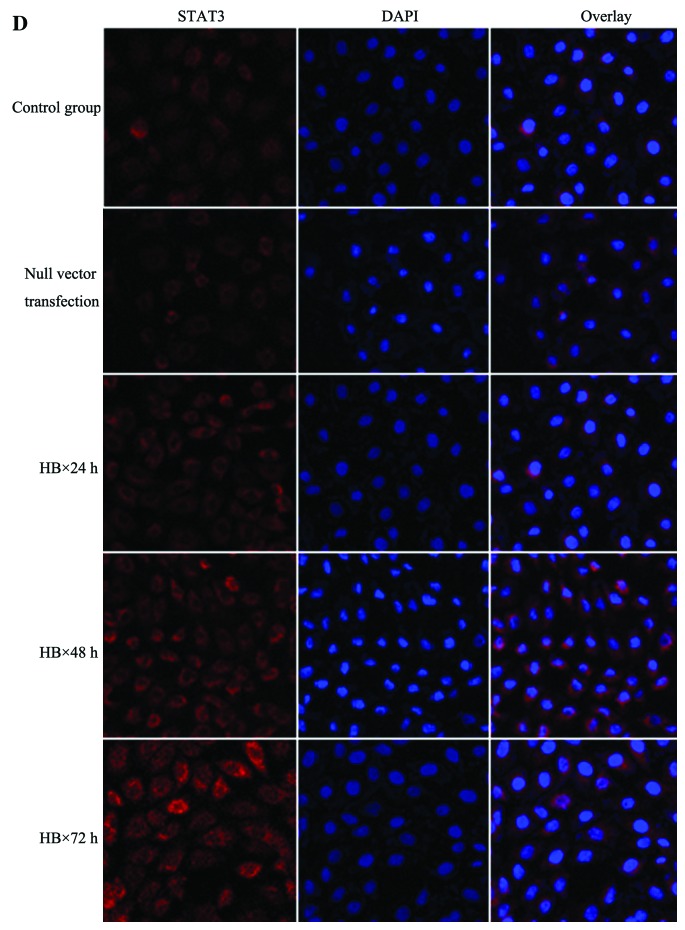

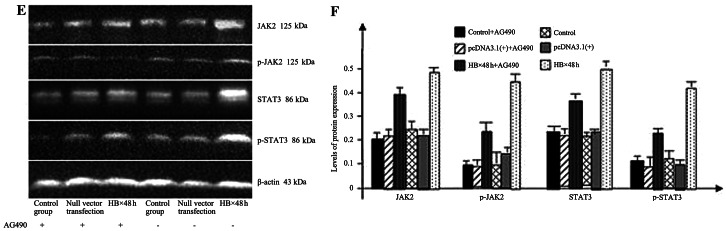
(A) Western blot bands for JAK2, p-JAK2, STAT3 and p-STAT3 expression in the control group, the pc-DNA3.1(+) group, the HBx 24 h group, the HBx 48 h group and the HBx 72 h group. (B) Histogram of the relative expression of JAK2, p-JAK2, STAT3 and p-STAT3 protein in the control group, the pc-DNA3.1(+) group, the HBx 24 h group, the HBx 48 h group and the HBx 72 h group. (C) Immunofluorescence showing p-STAT3 expression in the control group, the pc-DNA3.1(+) group, the HBx 24 h group, the HBx 48 h group and the HBx 72 h group. (D) Immunofluorescence showing STAT3 expression in the control group, the pc-DNA3.1(+) group, the HBx 24 h group, the HBx 48 h group and the HBx 72 h group (x400). (E) Western blot bands of JAK2, p-JAK2, STAT3 and p-STAT3 in the various groups with AG490, compared with the corresponding groups without AG490. (F) Histogram of the relative expression of JAK2, p-JAK2, STAT3 and p-STAT3 protein in the various groups with AG490, compared with the corresponding groups without AG490.

Western blot analysis revealed that JAK2 and STAT3 were expressed in the HK-2 cells of the control group, and slight expression of phosphorylated JAK2 and STAT3 was evident. No significant difference (P>0.05) was noted between the pc-DNA3.1(+) group and the control group. From 24 h after pcDNA3.1(+) HBx transfection, p-JAK2 and p-STAT3 expression obviously increased and respectively reached peak values at 72 h. After pc-DNA3.1(+) HBx transfection, significant differences in p-JAK2 and p-STAT3 expression among the various transfection groups and the control group (all P<0.05) were noted. JAK2 and STAT3 expression was also obviously increased at 24 h. With an increase in transfection time, protein expression demonstrated a gradual increasing trend and reached a peak at 72 h. Among the various transfection and control groups, significant differences (P<0.05) were achieved (Fig. 4A and B).

Cell immunofluorescence results showed that two types of signaling proteins (STAT3 and p-STAT3) were expressed in the various groups of renal tubular epithelial cells. In the control group, STAT3 and p-STAT3 proteins were mainly expressed in the cytoplasm, and p-STAT3 protein expression was lower. After pc-DNA3.1-HBx transfection, p-STAT3 protein levels in the various transfection groups were obviously increased and enhanced with the extension of time. At 72 h, the p-STAT3 protein level was highest, and higher expression in both the cytoplasm and nucleus was noted. At 24 h after pc-DNA3.1(+) HBx transfection, STAT3 protein level was obviously increased and was enhanced with the extension of time. At 72 h, it was highest, and STAT3 expression was mainly noted in the cytoplasm and nucleus (Fig. 4C and D).

Compared with the HBx 48 h group without AG490, p-JAK2, p-STAT3, JAK2 and STAT3 expression in the HBx 48 h group with AG490 was significantly reduced (P<0.05). Among the control group, the pc-DNA3.1(+) group, the control group with AG490 and the pc-DNA3.1(+) group with AG490, no significant difference was noted for expression of the above-menitoned four signaling proteins (P>0.05) (Fig. 4E and F).

### Bax/Bcl-2 ratio

**Figure 5:**
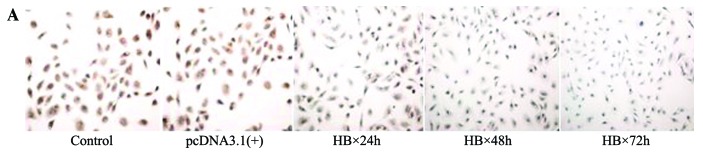

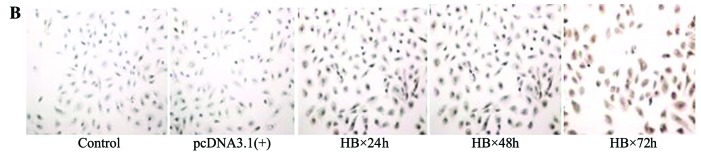

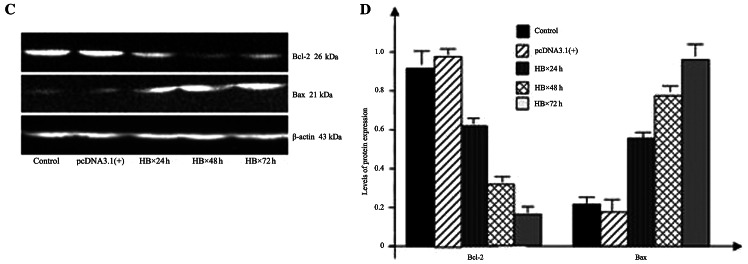

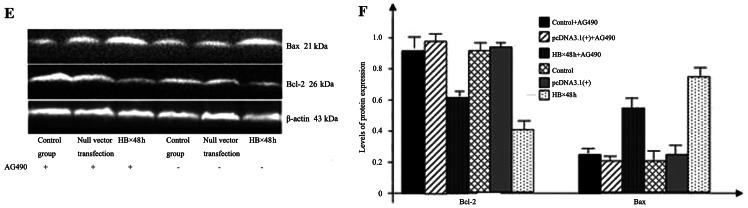
(A) Immunohistochemical analysis of Bcl-2 in the control group, the pc-DNA3.1(+) group, the HBx 24 h group, the HBx 48 h group and the HBx 72 h group (x200). (B) Immunohistochemical analysis of Bax in the control group, the pc-DNA3.1(+) group, the HBx 24 h group, the HBx 48 h group and the HBx 72 h group (x200). (C) Western blot bands of Bcl-2 and Bax in the control group, the pc-DNA3.1(+) group, the HBx 24 h group, the HBx 48 h group and the HBx 72 h group. (D) Histogram of the relative expression of Bcl-2 and Bax proteins in the control group, the pc-DNA3.1(+) group, the HBx 24 h group, the HBx 48 h group and the HBx 72 h group. (E) Western blot bands of Bcl-2 and Bax of various groups with AG490, compared with corresponding groups without AG490. (F) Histogram of the relative expression of Bcl-2 and Bax proteins in the various groups with AG490, compared with the corresponding groups without AG490.

Bcl-2 expression was present in the various groups of cells. The Bcl-2 expression level was higher in both the control group and the pc-DNA3.1(+) group, but no significant difference between the two groups (P>0.05) was achieved. At 24 h after pc-DNA3.1(+) HBx transfection, the Bcl-2 expression level began to decrease and declined to a minimum level at 72 h. Among the various pc-DNA3.1(+) HBx transfection groups and the control group, significant differences (P<0.05) were noted (Fig. 5A). Bax expression level was lower in both the control group and the pc-DNA3.1(+) group, but no significant difference between the two groups (P>0.05) was noted. At 24 h after pc-DNA3.1(+) HBx transfection, the Bax expression level began to increase and rose to the maximum level at 72 h. Among the various pc-DNA3.1(+) HBx transfection and control groups, significant differences (P<0.05) were noted (Fig. 5B).

Bcl-2 expression was determined by western blot analysis. The Bcl-2 expression level was higher in both the control group and the pc-DNA3.1(+) group, but no significant difference was noted between the two groups (P>0.05). At 24 h after pc-DNA3.1(+) HBx transfection, the Bcl-2 expression level began to decrease and declined to a minimum level at 72 h. Among the various pc-DNA3.1(+) HBx transfection and control groups, significant differences (P<0.05) were noted (Fig. 5C and D). The Bax expression level was lower in both the control group and the pc-DNA3.1(+) group, but no significant difference was noted between the two groups (P>0.05). At 24 h after pc-DNA3.1(+) HBx transfection, the Bax expression level began to increase and rose to a maximum level at 72 h. Among the various pc-DNA3.1(+) HBx transfection and the control groups, significant differences (P<0.05) were noted (Fig. 5C and D).

Between the control group and the control group with AG490 and between the pc-DNA3.1(+) group and the pc-DNA3.1(+) group with AG490, no significant difference (P>0.05) was noted. Bcl-2 expression level in the HBx transfection group obviously decreased. After AG490 was added, the Bcl-2 level slightly increased, but was significantly lower than that of the control group, and a significant difference (P<0.05) was noted (Fig. 5E and F). The Bax expression level was lower in the control group, the pc-DNA3.1(+) group, the control group with AG490 and the pc-DNA3.1(+) group with AG490. The Bax expression level in the HBx transfection group significantly increased, while the Bax expression level of the HBx transfection group with AG490 was significantly lower compared to the levels in the HBx transfection group, and significant differences (P<0.05) were noted (Fig. 5E and F).

### HK-2 cell proliferation

**Figure 6:**
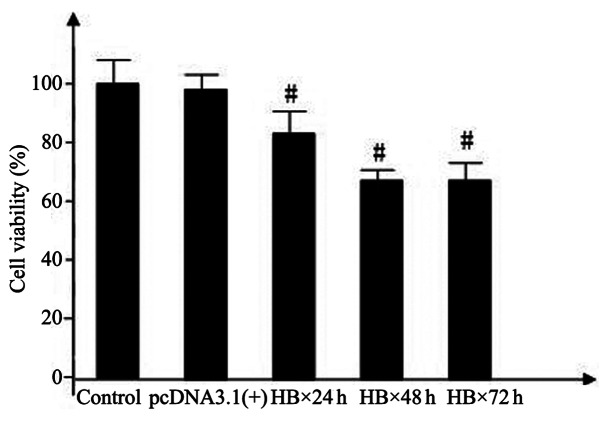
Cellular survival rates of the control, the pc-DNA3.1(+) group, the HBx 24 h, the HBx 48 h and the HBx 72 h groups.

The cellular survival rate of the control group was set as 100%. No significant difference in cellular survival rate (P>0.05) was noted between the pc-DNA3.1(+) and the control groups, indicating that pc-DNA3.1(+) did not obviously inhibited cellular proliferation. At 24 h after pc-DNA3.1(+) HBx transfection, the cellular survival rate obviously decreased, indicating that HBx had an inhibitory effect on cellular proliferation. The inhibition ratio was 14.08±2.14%. Compared with the control group, the difference was significant (P<0.05). At 48 h after pc-DNA3.1(+) HBx transfection, the inhibition ratio of cellular proliferation was 31.33±2.24%. Compared with the control and the HBx 24 h groups, both differences were significant (P<0.05). At 72 h, the inhibition ratio of cellular proliferation was 35.27±1.10%. Compared with the control and the HBx 24 h groups, both differences were significant (P<0.05), but compared with the HBx 48 h group, the difference was not significant (P>0.05) (Fig. 6).

### HK-2 cell apoptosis

**Figure 7:**
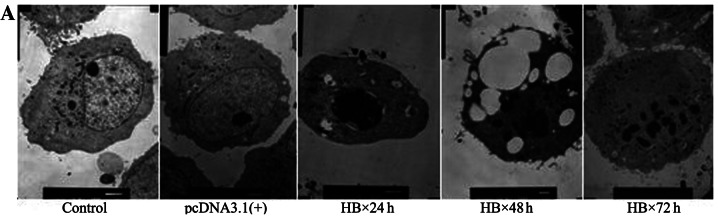

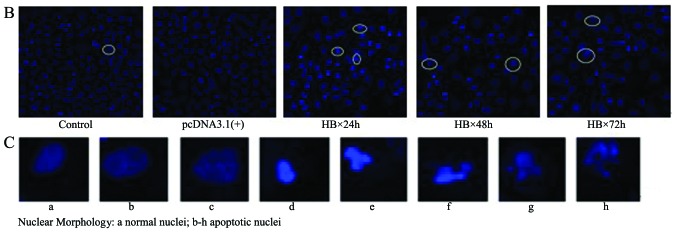

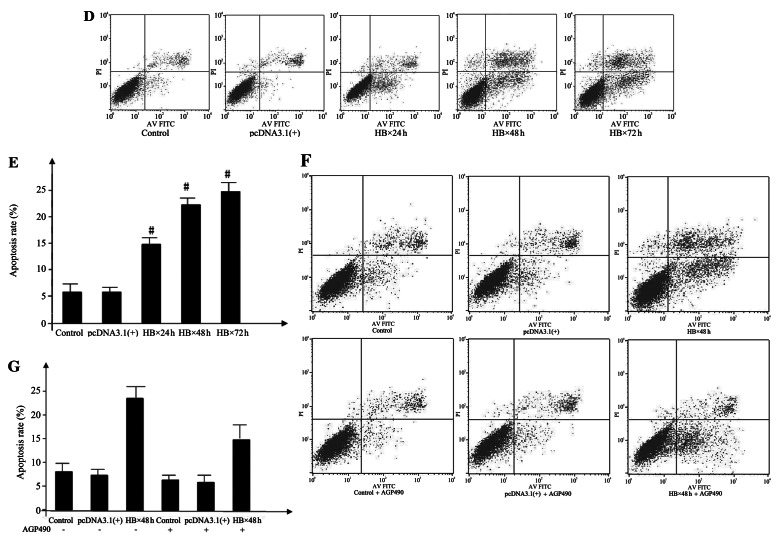
(A) Ultrastructures of the cells of the various groups as observed using transmission electron microscopy. (B) HO33342 staning revealed apoptotic morphologic variations in the cells of the various groups. The white ovals indicate typical apoptotic nuclear changes. (C) Typical apoptotic nuclei indicated by the white ovals in B were magnified. (D) Apoptosis rates in the various groups of cells as determined by flow cytometry. (E) Comparisons of the apoptosis rates in the cells of the various groups. (F) Changes in the cellular apoptosis rates in the various cell groups with AG490, compared with corresponding groups without AG490. (G) Histogram of the cellular apoptosis rate change in the various cell groups with AG490, compared with the corresponding groups without AG490.

HO33342, transmission electron microscopy and flow cytometry were used to determine the apoptosis in HK-2 cells after pcDNA3.1(+) and pcDNA3.1(+) HBx transfection. Transmission electron microscopy (Fig. 7A) was used to observe the ultrastructure of the cells of the various groups. In the control and the pc-DNA3.1(+) groups, nuclei were nearly circular, and plasmosomes were offset. The nuclear membrane was clear; there was a large amount of ribosomes and mitochondria and rough endoplasmic reticula in the cytoplasm, and microvilli were visible on the cell surface. After target gene HBx transfection, irregular and depressed plasmosomes, nuclear membrane swelling and intranuclear chromatin margination were visible in the HBx 24 h, HBx 48 h and HBx 72 h groups. At the advanced stage of apoptosis, nuclear membrane dissolutions and nuclear fragmentations were visible. After HO33342 staining, apoptotic morphological variations in the cells of the various groups after transfection were observed (Fig. 7B). In the control and pc-DNA3.1(+) groups, the nuclei of the HK-2 cells had clear outlines, the nuclei were normal, and nuclear fragmentations were visible. In the HBx 24 h, HBx 48 h and HBx 72 h groups, nuclear fragmentations and pyknoses were visible, and partial nuclei presented dissolutions and chromatin margination. With the extension of time, the cell count was significantly decreased, and nuclear variations were aggravated. Fig. 7B (white oval) shows various typical apoptotic nuclei, and Fig. 7B is amplified in Fig. 7C.

We further used flow cytometry to quantify the influence of the HBx gene on HK-2 cell apoptosis. After Annexin V-FITC/PI staining, the control, pc-DNA3.1(+), HBx 24 h, HBx 48 h and HBx 72 h groups were assessed by flow cytometry to analyze the percentage of cell apoptosis. The experiment was repeated in triplicate, and the results are expressed as means ± standard deviation. The apoptosis rate of HK-2 cells in the control group was 5.81±1.62%, the pc-DNA3.1(+) group was 5.92±1.09%, the HBx 24 h group was 15.18±1.98%, the HBx 48 h group was 20.62±2.23%, and the HBx 72 h group was 23.83±2.16%. No significant difference was noted between the pc-DNA3.1(+) and control groups. Cell apoptosis rate of the HBx 24 h group was significantly higher than that of the control group, and a significant difference between the two groups (P<0.05) was noted. Cell apoptosis rate of the HBx 48 h group was significantly higher than that of the HBx 24 h group, and a significant difference between the two groups (P<0.05) was noted. No significant difference (P>0.05) was noted between the HBx 48 h group and the HBx 72 h group (Fig. 7D and E).

After AG490 was added for incubation, the cell apoptosis rates for the 6 groups were compared (Fig. 7F and G). The cell apoptosis rate of the control group was 8.62±1.58%, the pc-DNA3.1(+) group was 8.34±1.25%, the control group with AG490 was 6.30±2.01%, and the pc-DNA3.1(+) group with AG490 was 7.39±1.74%. Between the control group and the control group with AG490 and between the pc-DNA3.1(+) group and the pc-DNA3.1(+) group with AG490, there was no significant difference in the cell apoptosis rate (both P>0.05). Cell apoptosis rate of the HBx 48 h group significantly increased to 23.85±1.42%. After AG490 was added for incubation, the cell apoptosis rate significantly decreased to 14.34±2.63%, but it was still higher than that of the control group. Compared with the control group, the difference was significant (P<0.05).

## Discussion

HBVGN has been recognized as the most prevalent extrahepatic lesion caused by HBV infection, and, to date, its pathogenesis has not been completely clarified. In recent years, more research has focused on the existence and significance of HBV-related nucleic acid molecules in nephridial tissue. It was found that there are HBV-DNA, HBV-RNA and closed circular double-chain DNA (cccDNA) and even complete virus particles (21–23) in the kidneys of patients with HBVGN, which further supports the viewpoint that HBV can directly infect the kidney and *in situ* reproduction to cause diseases.

Clippinger *et al*(4,24) used primary-cultured liver cells to successively research the location of HBx protein in the mitochondrion and its influence on liver cell apoptosis. As a result, it was found that in liver cells, HBx not only inhibits apoptosis, but also stimulates apoptosis, mainly depending on the NF-κB signaling pathway status. However, in renal tubular epithelial cells, the correlation between the HBx protein and renal tubular epithelial cell apoptosis and its signaling pathway are still unclear to date.

Apoptosis is one type of programmed cell death caused by the intracellular pre-stored death program triggered by internal and external factors and is a complex process involving extracellular signaling and intracellular signaling pathway apoptosis-related genes and enzymes (25). The regulation of various extracellular factors on cell behavior is closely related to the activation of intracellular multiple signaling transduction pathways. Among them, activation of the JAK/STAT pathway in cell apoptosis has been widely studied in recent years (26–28). The JAK/STAT signaling pathway is one of the important pathways of cytokine transduction and can play an important regulatory role in cellular physiological and pathological reactions. JAK is an endogenous protein tyrosine kinase, and it can be activated following the binding of the cytokine receptor with the corresponding aglucon to cause phosphorylation of the STAT molecule in the cytoplasm. Two phosphorylated STAT molecules form a dimer to enter the nucleus, and they bind with the specific DNA sequence of the target gene promotor in the nucleus to induce target gene expression. Among them, STAT tyrosine phosphorylation is the key link of the JAK/STAT signaling pathway regulating transcription and exerting multiple biological effects (29).

Our clinical pathological study showed that, compared with the control group, p-STAT3 signal protein expression in HBVGN nephridial tissues was significantly increased, especially in renal tubular epithelial cells. To further verify the correlation between the JAK2/STAT3 signaling pathway and the HBx protein, we infected *in vitro* cultured renal tubular epithelial cells (HK-2 cell strain) with the HBx gene and used immunocytochemical and western blot analyses to determine the activation of the JAK2/STAT3 signaling pathway. The results showed that HBx increased p-STAT3 expression and activated the JAK2/STAT3 signaling pathway *in vitro*, which was in line with an increase in p-STAT3 expression in nephridial tissues of patients with HBVGN. It was further confirmed that JAK2/STAT3 signaling pathway activation is involved in the occurrence and development of HBVGN.

Deng *et al*(30,31) used HBV-DNA-positive serum to culture human renal tubular epithelial cells *in vitro* and found that HBV-DNA induced cell apoptosis, and the cell apoptosis ratio was positively related to the HBV-DNA level. Uncontrollable renal tubular epithelial cell apoptosis caused renal injury and thus resulted in interstitial fibrosis (32,33). In the present study, we further proved that the pathological change in renal tubular epithelial cell apoptosis caused by HBx protein expression upregulation after HBV infection is one of the pathogeneses of HBVGN. Cell culture *in vitro* and plasmid transfection further confirmed that HBx can cause HK-2 cell apoptosis and this cell apoptosis is related to the JAK2/STAT3 signaling pathway activation. Recently, some researchers believe that cell density or fusion status influences the biological behaviors and responses of different cells, including cell apoptosis (34,35). In this study, all experiments were completed under a constant cell subfusion status (~70% fusion), and a control group transfected with empty vector was established. Therefore, we believe that the obtained results are credible.

For further confirmation that JAK2/STAT3 signaling pathway activation is involved in renal tubular epithelial cell apoptosis, we used the JAK/STAT signaling pathway blocker AG490. AG490 is a specific inhibitor of JAK2, and effectively inhibits JAK2 tyrosine phosphorylation and blocks its downstream signal transduction and activation of transcription activation factor STAT (36). After AG490 treatment, the increased HK-2 cell apoptosis rate induced by HBx gene transfection significantly declined. At the same time, p-JAK2 and p-STAT3 expression slightly decreased. These results suggest that JAK/STAT signaling pathway activation is involved in renal injury caused by HBV infection.

Previous studies have shown that an increased ratio of apoptosis-related proteins Bax/Bcl-2 is involved in the occurrence of cell apoptosis (37–39). In this study, Bcl-2 expression decreased with JAK2/STAT3 signaling pathway activation after HBx gene transfection, whereas Bax expression increased and the Bax/Bcl-2 ratio significantly rose. Application of the JAK2/STAT3 signaling pathway blocker AG490 partially blocked the activation of the JAK2/STAT3 signaling pathway and reduced the Bax/Bcl-2 ratio and finally caused a significant decrease in the cell apoptosis rate. As an upstream kinase inhibitor of STAT3 activation, AG490 reduces cell apoptosis by inhibiting the JAK2/STAT3 signaling pathway. The above results suggest that the change in Bax/Bcl-2 ratio was involved in the process of renal tubular epithelial cell apoptosis caused by HBx and regulated by JAK2/STAT3 signaling pathway activation.

In conclusion, our study suggests that activation of the JAK2/STAT3 signaling pathway and increase in the Bax/Bcl-2 ratio are involved in the process of renal tubular epithelial cell apoptosis caused by HBx. This finding further supports the theory that HBV directly damages nephridial tissues in HBVGN and provides an experimental basis and a theoretical basis for establishing a novel clinical treatment mode - signal pathway-blocking treatment. Since an immortalized cell strain was used as the object of investigaion in the present study, it is necessary to conduct further research.
